# Outcomes for Māori clients in the Housing First programme in Aotearoa New Zealand.

**DOI:** 10.23889/ijpds.v7i3.1834

**Published:** 2022-08-25

**Authors:** Saera Chun, Jenny Ombler, Tiria Pehi

**Affiliations:** 1 University of Manitoba

## Objectives

We present the backgrounds and outcomes for formerly homeless Māori (indigenous to Aotearoa) clients of the first Housing First programme in Aotearoa New Zealand, delivered by The People’s Project in Kirikiriroa. We compare the result from the Housing First Māori clients to the non-Māori comparison group and the general population.

## Approach

The Integrated Data Infrastructure (IDI) is linked microdata administered by Tatauranga Aotearoa Statistics New Zealand. It enables a comprehensive picture of the life course and outcomes for people who have received an intervention such as Housing First. The People’s Project supplied a list of identifiers of the Housing First clients who were supported into permanent housing between 2014 and 2017. De-identified data from clients were linked to a central dataset in the IDI. We investigated service interaction histories, and up to four years post-housing outcomes across a broad range of datasets, including health, justice, social development, and income data.

## Results

Comparing Māori clients to non-Māori demonstrated the existing systematic barriers that prevent access to services and result in worse outcomes for general Māori population also persist for the formerly homeless Māori. While all clients had higher service usage rates compared to the general population before being housed and continue to do so after being housed, Māori still faced barriers that their service usages rates were lower and outcomes were less favourable than non-Māori. This was especially visible in health datasets.

Māori clients have higher charge rates per offending on average. While victimisation rates increased for everyone after being housed, Māori clients were more likely to be crime victims. Income analysis showed that while Māori clients were employed at similar rates, they earned less than non-Māori.

## Conclusion

We demonstrated that interventions like Housing First are positive, but when implemented within an inequitable system for certain populations, can be less effective for those populations. Programmes to address homelessness should be implemented in the context of wider systems change that addresses the root causes of inequities to be effective.

